# The cirrhotic liver is depleted of docosahexaenoic acid (DHA), a key modulator of NF-κB and TGFβ pathways in hepatic stellate cells

**DOI:** 10.1038/s41419-018-1243-0

**Published:** 2019-01-08

**Authors:** Mónica Enguita, Nerea Razquin, Reinald Pamplona, Jorge Quiroga, Jesús Prieto, Puri Fortes

**Affiliations:** 1 Department of Gene Therapy and Hepatology, Center for Applied Medical Research (CIMA), University of Navarra (UNAV), Pamplona, Spain; 2 Navarra Institute for Health Research (IdiSNA), Pamplona, Spain; 3 Department of Experimental Medicine, University of Lleida (IRB), Lleida, Spain; 4 Liver Unit, Clínica Universidad de Navarra, Pamplona, Spain; 5 Centro de Investigación Biomédica en Red de Enfermedades Hepáticas y Digestivas (Ciberehd), Pamplona, Spain; 6 CIMA/UNAV, Pamplona, Spain

## Abstract

Liver cirrhosis results from chronic hepatic damage and is characterized by derangement of the organ architecture with increased liver fibrogenesis and defective hepatocellular function. It frequently evolves into progressive hepatic insufficiency associated with high mortality unless liver transplantation is performed. We have hypothesized that the deficiency of critical nutrients such as essential omega-3 fatty acids might play a role in the progression of liver cirrhosis. Here we evaluated by LC-MS/MS the liver content of omega-3 docosahexaenoic fatty acid (DHA) in cirrhotic patients and investigated the effect of DHA in a murine model of liver injury and in the response of hepatic stellate cells (HSCs) (the main producers of collagen in the liver) to pro-fibrogenic stimuli. We found that cirrhotic livers exhibit a marked depletion of DHA and that this alteration correlates with the progression of the disease. Administration of DHA exerts potent anti-fibrogenic effects in an acute model of liver damage. Studies with HSCs show that DHA inhibits fibrogenesis more intensely than other omega-3 fatty acids. Data from expression arrays revealed that DHA blocks TGFβ and NF-κB pathways. Mechanistically, DHA decreases late, but not early, SMAD3 nuclear accumulation and inhibits p65/RelA-S536 phosphorylation, which is required for HSC survival. Notably, DHA increases *ADRP* expression, leading to the formation of typical quiescence-associated perinuclear lipid droplets. In conclusion, a marked depletion of DHA is present in the liver of patients with advanced cirrhosis. DHA displays anti-fibrogenic activities on HSCs targeting NF-κB and TGFβ pathways and inducing *ADPR* expression and quiescence in these cells.

## Introduction

Persistent liver damage causes repeated boosts of cell death and regeneration, ultimately leading to a distortion of liver architecture known as liver cirrhosis. Cirrhosis is characterized by the formation of regenerative hepatocellular nodules surrounded by a fibrous tissue. Hepatocellular dedifferentiation and increased collagen deposition by activated hepatic stellate cells (HSCs) constitute the pathophysiologic landmark of liver cirrhosis^1^. This condition is associated with defective liver function and increased portal pressure and tends to progress to stages of more severe hepatic insufficiency and portal hypertension, resulting in complications such as portal systemic encephalopathy, gastrointestinal bleeding, malnutrition and ascite formation.

The characterization of factors that might promote cirrhosis progression is of considerable clinical interest. We have reasoned that in advanced liver cirrhosis a number of disturbances such as porto-systemic shunting, inadequate dietary intake, impaired intestinal absorption, compromised integrity of the intestinal barrier, inflammation and altered metabolism might deprive liver cells of essential nutrients^2,3^, and that this defect could potentially influence the evolution of the disease.

Omega-3 polyunsaturated fatty acids (ω3-PUFAs) are essential fatty acids that cannot be synthesized by the body and need to be obtained from the diet. The most relevant ω3-PUFA compounds are eicosapentaenoic acid (EPA), docosahexaenoic acid (DPA) and docosahexaenoic acid (DHA). These molecules display potent anti-inflammatory effects in different conditions by inhibiting leukocyte chemotaxis and blocking the production of eicosanoids and pro-inflammatory cytokines^4^. Moreover, ω3-PUFAs impact cell function by influencing cell membrane composition, disrupting lipid rafts, activating G-coupled protein receptors, acting as precursors of pro-resolving mediators and modulating key transcription factors such as PPARs, SREBP1, ChREB, NF-κB and LXR^5^. Accordingly, hepatic DHA abundance may likely modulate the liver response to injury. However, although some studies in cirrhotic patients have shown a reduction in the circulating levels of ω3-PUFA^6,7^, there is no information at all regarding DHA values in the cirrhotic liver, the site where this omega-3 would display its tissue homeostatic effects.

Fat-1 transgenic mice (which are capable of producing ω3-PUFA from ω6-PUFA) are protected against multiple liver insults such as alcohol and high-fat diet (HFD), non-alcoholic steatohepatitis (NASH) and DEN-induced HCC^8–11^. Moreover, oral administration of ω3-PUFA displays protective activities in several animal models of chronic hepatic damage, including NASH and CCl_4_-induced liver fibrosis. In these conditions ω3-PUFAs decrease steatosis, inflammation and fibrosis^12–15^. The anti-fibrotic effect of these compounds is in line with their ability to reduce *COL1A1* expression by TGFβ-stimulated HSCs^14^. However, the molecular mechanisms underlying this effect remain elusive.

In the present study, we show that the cirrhotic liver is strikingly depleted of DHA in parallel with the progression of the disease and that DHA reverts HSCs to quiescence and inhibits the fibrogenic responses to TGFβ stimulation and NF-κB activation by affecting SMAD3 nuclear accumulation and p65/RelA S536 phosphorylation.

## Results

### DHA is deficient in cirrhotic livers

**Fig. 1:**
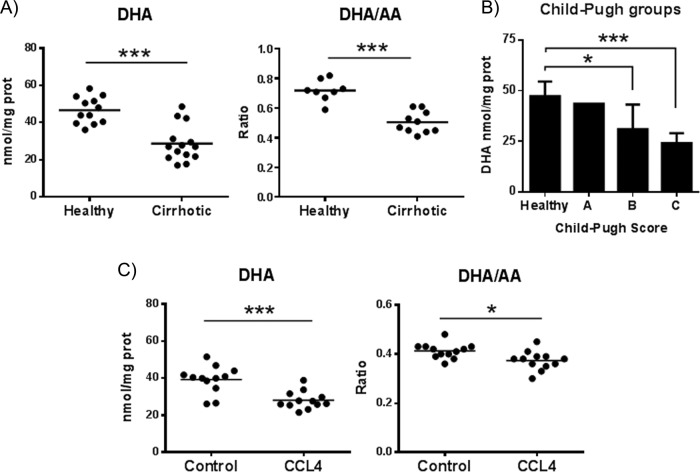
DHA levels decrease in cirrhotic livers. DHA and AA levels were determined by LC-MS/MS in liver samples from healthy and cirrhotic patients (**a**) or in liver tissue from control or CCl_4_-treated mice (**c**). **b** DHA levels in patients stratified according to Child-Pugh score

DHA has been reported to be decreased in the plasma of cirrhotic patients^6,7^, but there is no information concerning its values in the cirrhotic liver, a site where DHA biological effects could be crucial in maintaining tissue homeostasis. Thus, we used liquid chromatography-tandem mass spectrometry (LC-MS/MS) to determine DHA, DPA and arachidonic acid (AA) levels in liver samples from 14 cirrhotic patients and in 12 normal liver specimens (Supplementary table 1). We found that DHA, but not AA or DPA, values were markedly reduced in the cirrhotic liver tissue (Fig. 1a and Supplementary Fig. 1). Accordingly, the ratio DHA/AA in hepatic tissue was significantly diminished in cirrhotic patients compared to controls (Fig. 1a). Interestingly, the stratification of the patients according to Child-Pugh score indicated that DHA abundance decreased in association with the progression of the disease (Fig. 1b). DHA and the ratio DHA/AA were also significantly decreased in fibrotic mouse livers compared to control livers (Fig. 1c). A similar decrease in hepatic DHA was also observed in TAA-treated fibrotic mice (*n* = 7; **p < 0.01; data not shown).

**Fig. 2:**
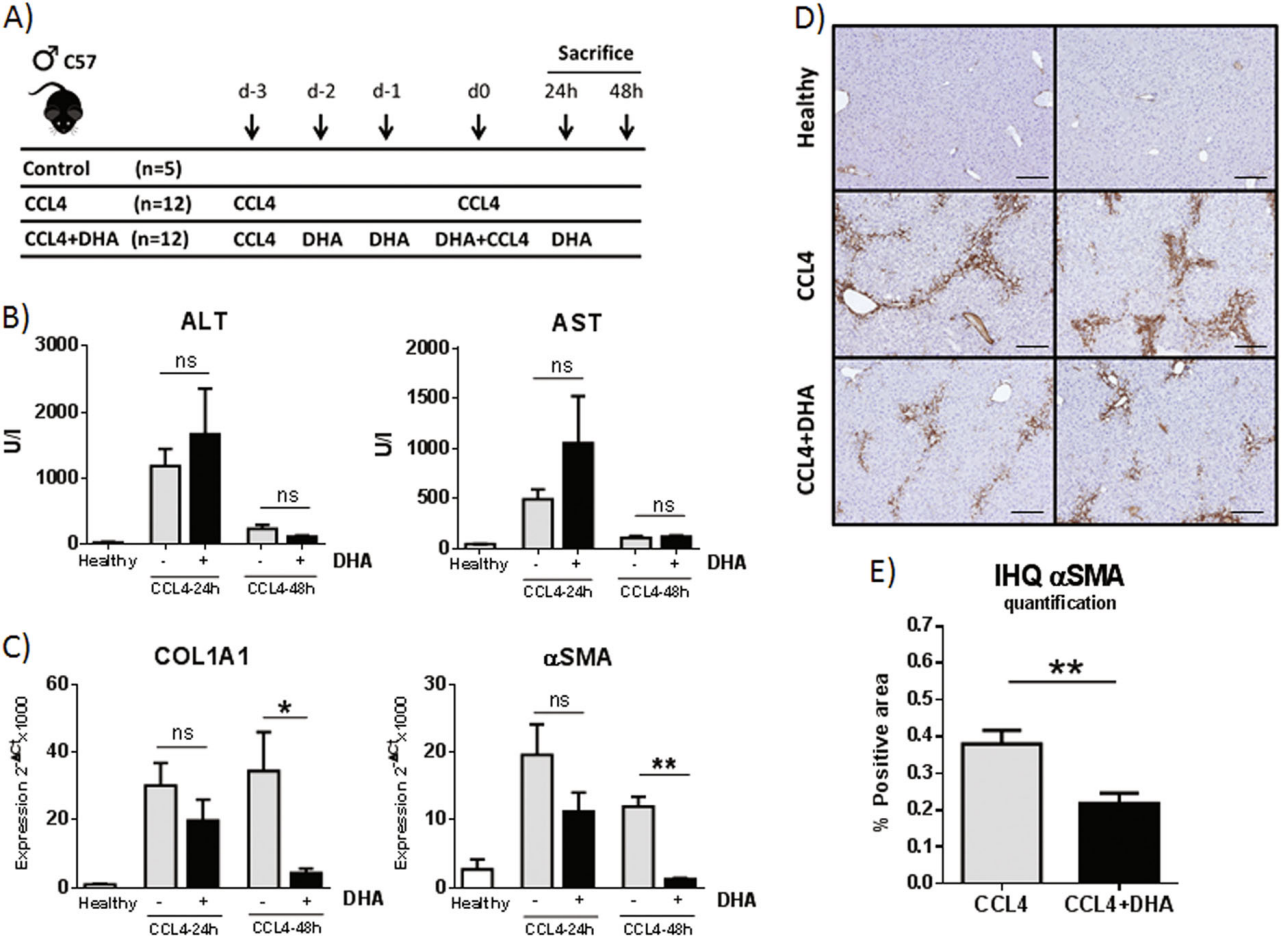
DHA has anti-fibrogenic effects in an acute liver damage model. **a** Schematic of the experiment. **b** Serum ALT and AST. **c** *COL1A1* and *αSMA* expression. **d** IHQ of αSMA in livers from mice sacrificed 48 h after the last dose of CCl_4_. Scale bar, 100 μm. **e** Quantification of the αSMA-positive area with ImageJ software. Results shown are from a representative experiment out of the three experiments performed

DHA has been shown to reduce oxidant stress and fibrogenesis in the liver in mouse models of NASH or chronic CCl_4_ administration^14,16^. Although DHA was found to inhibit TGFβ-induced *COL1A1* expression by human Lx2 stellate cells, the molecular mechanisms underpinning DHA anti-fibrogenic activity remain elusive. To better understand how DHA modulates liver fibrosis and the response to injury, we first studied the effect of DHA in a model of acute liver injury (Fig. 2a). Animals were challenged with CCl_4_ (days −3 and 0) and given by intragastric route (days −2, −1, 0 and + 1) DHA in sesame oil or vehicle alone, and were sacrificed at 24 and 48 h after the last CCl_4_ dose. At 24 h we found that hepatic DHA levels were increased in those animals given DHA but serum transaminases (ALT and AST), serum bilirubin and hepatic *COL1A1*, and *αSMA* mRNA levels showed similar values in both DHA-treated and -untreated mice, indicating comparable tissue damage in the two groups and no significant effect of DHA therapy at that time (Fig. 2b, c and data not shown). However, one day later, DHA-treated mice showed a marked reduction of *COL1A1* and *αSMA* mRNAs and a diminished number of αSMA-positive cells compared to controls (Fig. 2c–e). These data suggest that DHA does not prevent hepatocellular damage but greatly attenuates the activation and fibrogenic response of HSCs in this model. Therefore, we proceeded to explore the biological effects of DHA on this cell population.

### DHA exceeds other ω3-PUFAs in its ability to inhibit HSC activation and proliferation and to induce HSC quiescence

**Fig. 3:**
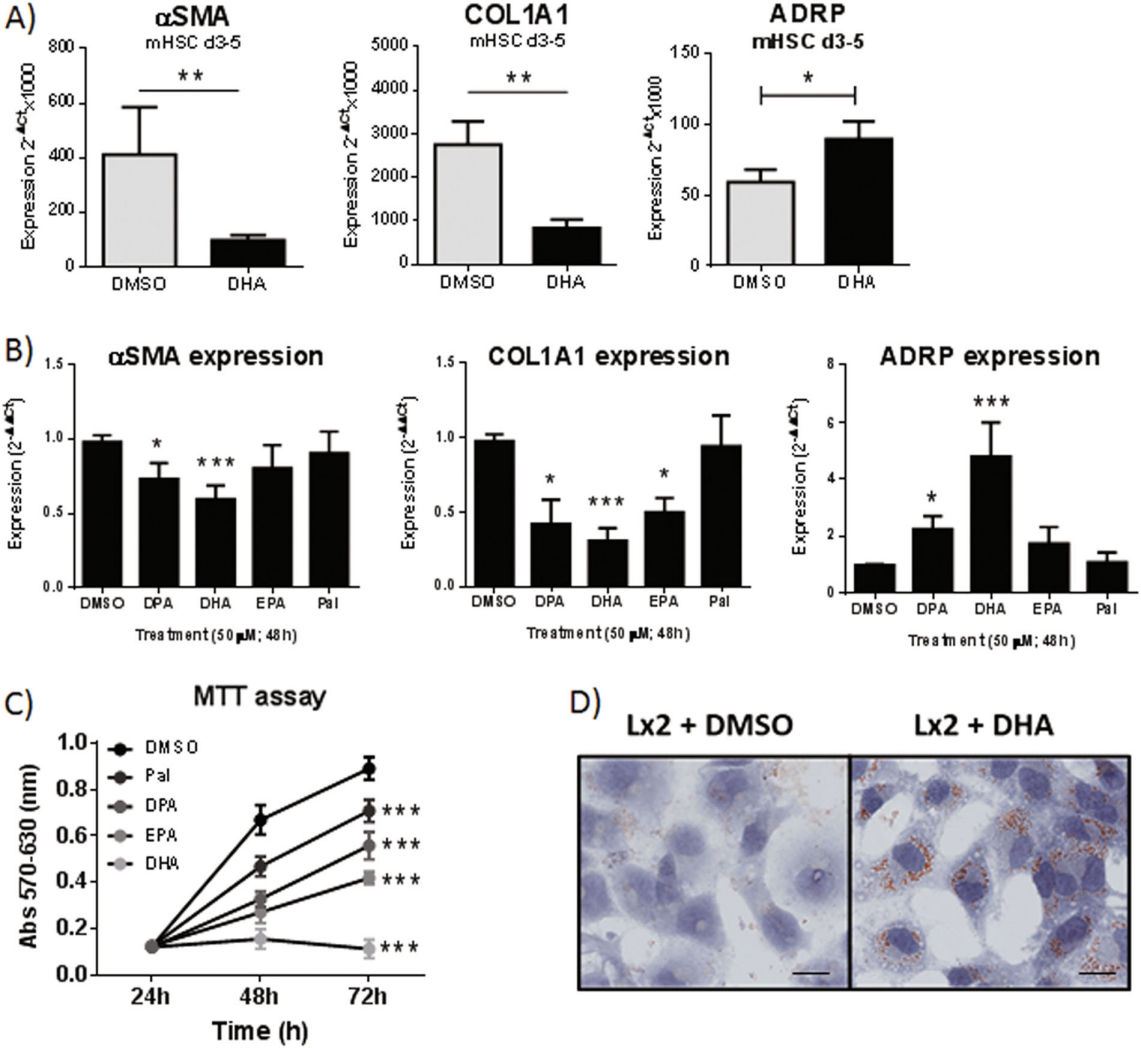
DHA induces stronger anti-fibrogenic effects than other FAs. **a** *αSMA*, *COL1A1* and *ADRP* expression in primary mHSCs plated for 3 days and treated with DMSO or 100 µM DHA for 48 h. **b–d** Lx2 cells treated with DMSO or with 50 µM of the indicated PUFAs. *αSMA*, *COL1A1* and *ADRP* expression at 24 h (**b**), MTT assay at indicated times (**c**) and lipid droplet content at 72 h (**d**). Scale bar, 20 μm. Experiments were performed two (**a**, **b**) or three (**c**) times

In both human Lx2 stellate cells and primary mouse HSCs (mHSCs), we found that DHA reduced HSC proliferation and expression of pro-fibrogenic factors (*COL1A1*, *αSMA* and *PDGFRβ*) in a dose-dependent manner (Supplementary Fig. 2). In addition, DHA markedly enhanced the expression of *ADRP*, a molecule that promotes lipid droplet formation (Fig. 3a). Importantly, DHA was more efficient at inducing these effects than other ω3-PUFAs (Fig. 3b, c). DHA is metabolized to lipid mediators with anti-inflammatory properties, such as Maresin, Resolvin D1 or 17(R)-Resolving D1, an aspirin-triggered epimer of Resolvin D1. However, treatment of Lx2 cells with these mediators did not affect the levels of *COL1A1* or *αSMA* mRNAs (data not shown). As a whole, our results indicate that DHA is the strongest anti-fibrotic ω3-PUFA and that these effects could be exerted directly by DHA. Of note, Lx2 cells treated with DHA showed a marked enrichment in cytoplasmic lipid droplets, a characteristic feature of quiescent HSCs (Fig. 3d).

### Transcriptome analyses reveal that DHA modulates plural cell response pathways in both Lx2 cells and mHSCs

**Fig. 4:**
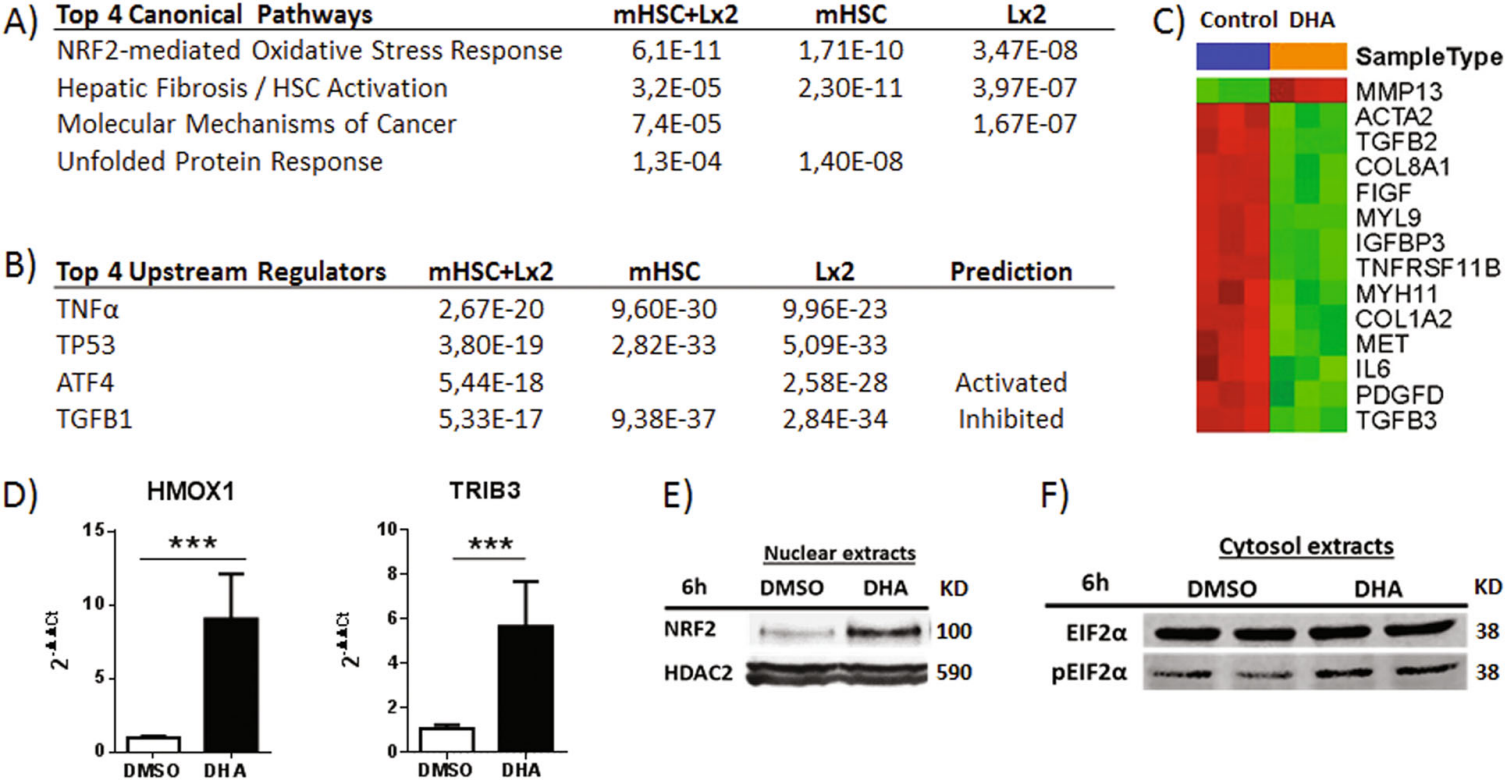
DHA affects several signaling pathways in Lx2 and mHSCs. **a,** **b** Differentially expressed genes (*B* > 0) from expression arrays were scrutinized by Ingenuity analysis to identify the altered top canonical pathways (**a**) and the top upstream regulators (**b**). The *p* value and the prediction state are indicated. **c** Heat map of the differentially expressed genes in mHSCs from the “Hepatic Fibrosis/Hepatic Stellate Cell Activation” pathway. **d**–**f** Lx2 cells were control-treated or incubated with 50 µM DHA. *HMOX1* and *TRIB3* expression after 48 h (**d**). Data are means ± SD from two experiments. Nuclear NRF2 accumulation after 6 h (**e**). EIF2α and phosphorylated EIF2α (pEIF2α) after 6 h (**f**)

Ingenuity pathway analysis of microarray data obtained from mHSCs and Lx2 cells indicated that the most highly affected processes in DHA-treated cells were HSC activation/hepatic fibrosis, NRF2-mediated oxidative stress response and ATF4-mediated unfolded protein response (UPR) (Fig. 4a). The most significant upstream regulators of DHA-altered genes were TGFβ (inhibited), ATF4 (activated), TP53 and TNFα (Fig. 4b). Thus, our data showed that DHA downregulated pro-fibrogenic factors (*COL1A1*, *αSMA*, *PDGFRβ* and *TGFβ*) and cell survival molecules (*cMET*) and upregulated anti-fibrogenic and cytoprotective factors (*HGF*) in both mHSCs (Fig. 4c) and Lx2 cells (Supplementary Fig. 3). In addition, DHA also enhanced the expression of NRF2 target genes (such as *HMOX1*) and ATF4-dependent genes (such as *TRIB3*) (Fig. 4d). Consistently, DHA-treated Lx2 cells increased eIF2α phosphorylation (an event occurring during UPR) and mobilization of NRF2 to cell nuclei (Fig. 4e, f). These observations led us to analyze whether the effects on NRF2 and UPR pathways were implicated in DHA anti-fibrogenic activity. With this aim, we overexpressed or knocked down (using siRNA) *NFR2* in Lx2 cells and analyzed the expression of *HMOX1*, *COL1A1* and *αSMA*. We found that *NFR2* overexpression enhanced *HMOX1* mRNA levels but did not affect *COL1A1* or *αSMA* expression. Similarly, *NRF2* knockdown did not attenuate DHA-mediated inhibition of pro-fibrogenic factors (Supplementary Fig. 4a–b). Furthermore, increasing eIF2α phosphorylation and thus ATF4 activation with salubrinal or inhibiting ATF4 activation with ISRIB did not affect the levels of *COL1A1* or *αSMA* mRNAs and did not alter the suppressive properties of DHA on *COL1A1* and *αSMA* expression (Supplementary Fig. 4c-d). These observations suggested that DHA anti-fibrogenic activity was not mediated by the NRF2 or ATF4 pathway. Therefore, we focused on the analysis of the effects of DHA on TGFβ and NF-κB pathways.

### DHA modulates nuclear SMAD3 accumulation and abrogates the induction of TGFβ delayed target genes

**Fig. 5:**
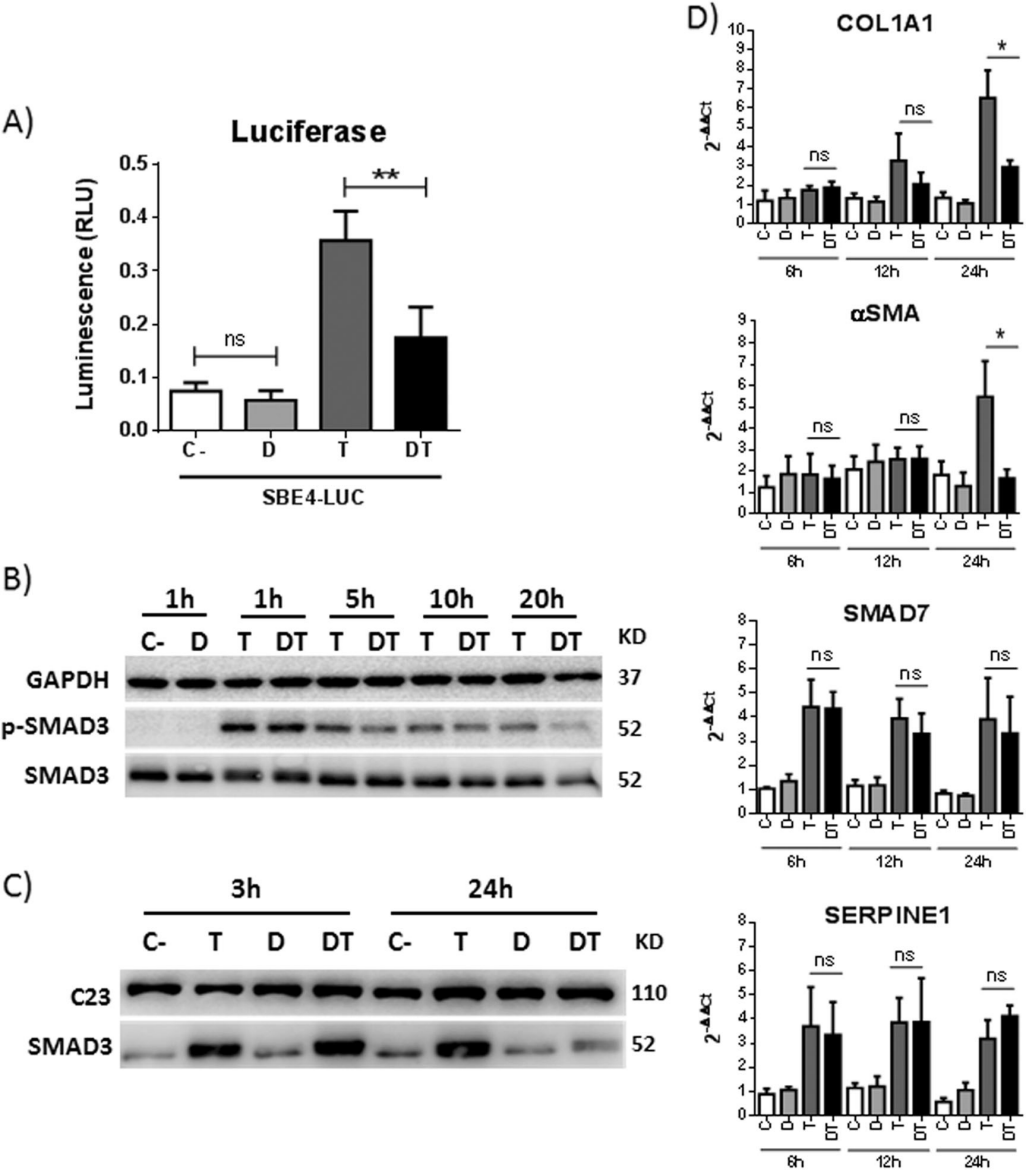
DHA decreases TGFβ-induced expression of *COL1A1* and *αSMA* by affecting the nuclear accumulation of SMAD3. Lx2 cells treated with DMSO (C-), 30 µM of DHA (D), 7.5 ng/ml TGFβ (T) or both DHA and TGFβ (DT) for the indicated times. **a** Luciferase levels measured 24 h after treatments in cells previously transfected with the SBE-Luc plasmid. Data are mean ± SD from two experiments. **b** GAPDH, SMAD3 and p-SMAD3 proteins in total extracts. **c** C23 and SMAD3 proteins in nuclear extracts. **d** *SERPINE1*, *SMAD7*, *COL1A1* and *αSMA* mRNA levels. Data are mean ± SD from two experiments

As mentioned, transcriptomic analyses suggested that DHA treatment of HSCs inactivates the TGFβ pathway. The TGFβ canonical pathway requires phosphorylation of the SMAD2/3 complex, binding to SMAD4 and nuclear translocation of the hetero-oligomeric complex. We observed that DHA blocked the production of luciferase from the SBE4-Luc plasmid, which requires SMAD3 for its expression (Fig. 5a). Kinetic experiments showed that TGFβ treatment induced the phosphorylation of SMAD3 and that DHA did not affect the total levels of SMAD3 or phosphorylated SMAD3 (p-SMAD3) up to 24 h post TGFβ treatment (Fig. 5b). However, while the nuclear levels of SMAD3 were similar between cells incubated and those not incubated with DHA at early times (3 h) post TGFβ treatment, at a later time (24 h), a drastic decrease in the amount of nuclear SMAD3 was observed in DHA-treated cells (Fig. 5c). In agreement with these results, the expression of targets induced at early times post TGFβ treatment, such as *SERPINE1/PAI-I* or *SMAD7*, was not affected by DHA, while delayed targets, such as *COL1A1* or *αSMA*, showed a highly significant decrease in DHA-incubated cells compared to controls (Fig. 5d). Similar results were observed at 24 and 48 h post TGFβ treatment (data not shown).

### DHA decreases phosphorylation and activity of the NF-κB subunit p65/RelA

**Fig. 6:**
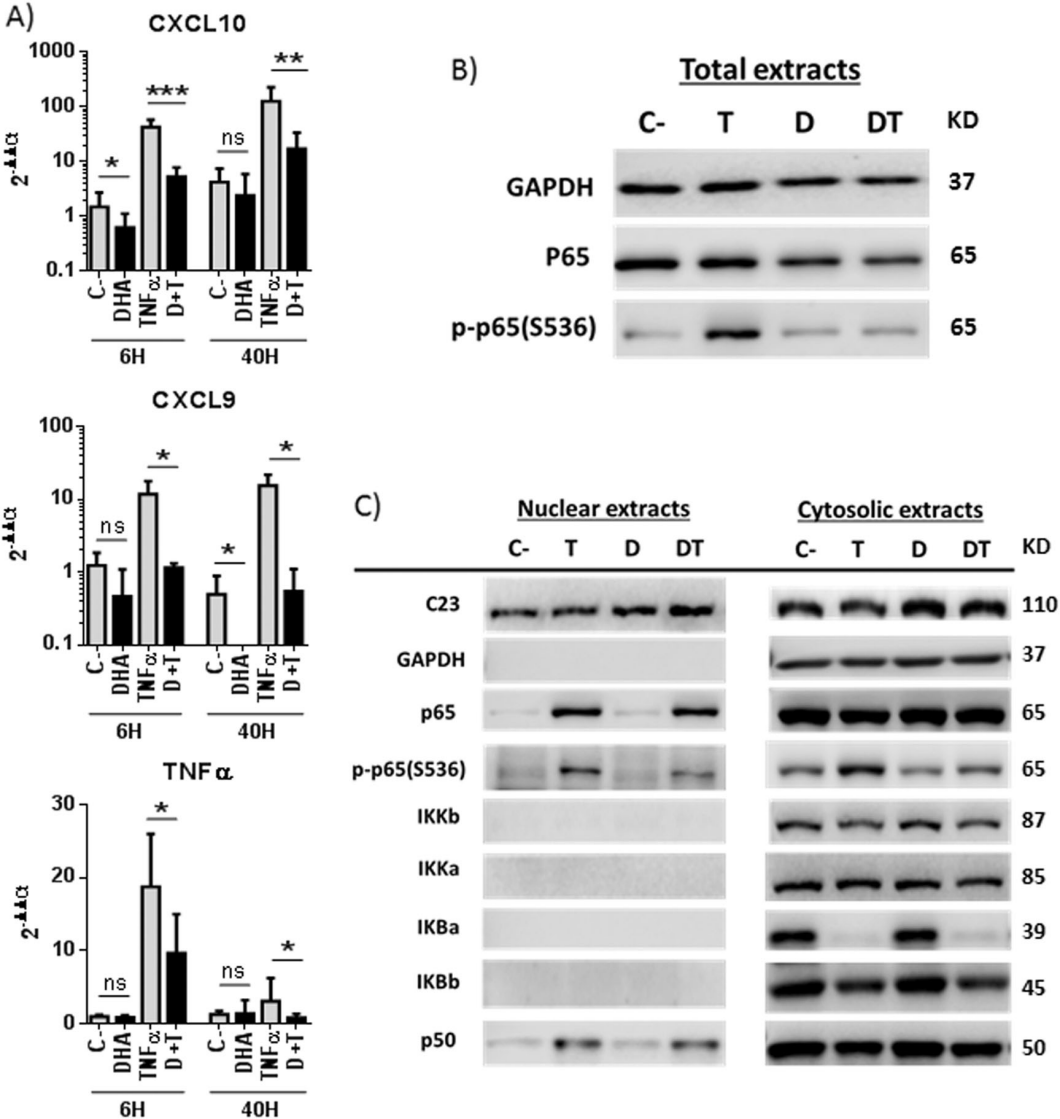
DHA decreases p65/RelA Ser^536^Ph. Lx2 cells were pretreated with DMSO or 30 µM DHA for 2 h and then incubated or not with 35 ng/ml TNFα for 15 min (**b**, **c**), 6 or 40 h (**a**). *CXCL10*, *CXCL9* and *TNFα* mRNA levels (**a**). Data are mean ± SD from three experiments. NF-κB signaling pathway proteins in total (**b**), nuclear and cytoplasmic (**c**) extracts. GAPDH and C23 were used as loading and fractionation controls

DHA modulates the NF-κB pathway in HSCs, as transcriptome analyses in DHA-treated cells showed TNFα as a top upstream regulator (Fig. 4). We observed that DHA drastically reduces the levels of pro-inflammatory transcripts *CXCL10*, *CXCL9* or *TNFα* in Lx2 cells stimulated with TNFα for 6 or 40 h (Fig. 6a). Using total extracts from cells treated with TNFα for 15 min, we observed that DHA does not affect the levels of the p65/RelA subunit of NF-κB, but drastically decreases p65/RelA serine 536 phosphorylation (p-p65(S536)) (Fig. 6b). When nuclear and cytoplasmic fractions were evaluated, we found that most components of the NF-κB pathway (IKKs, IκBα, IκBβ, p65, and p50) were not altered by DHA treatment (Fig. 6c). The exception was p-p65(S536), which decreased in both nuclear and cytoplasmic extracts of DHA-treated cells. Similar results were observed in cells treated with TNFα for 6 h (data not shown).

**Fig. 7:**
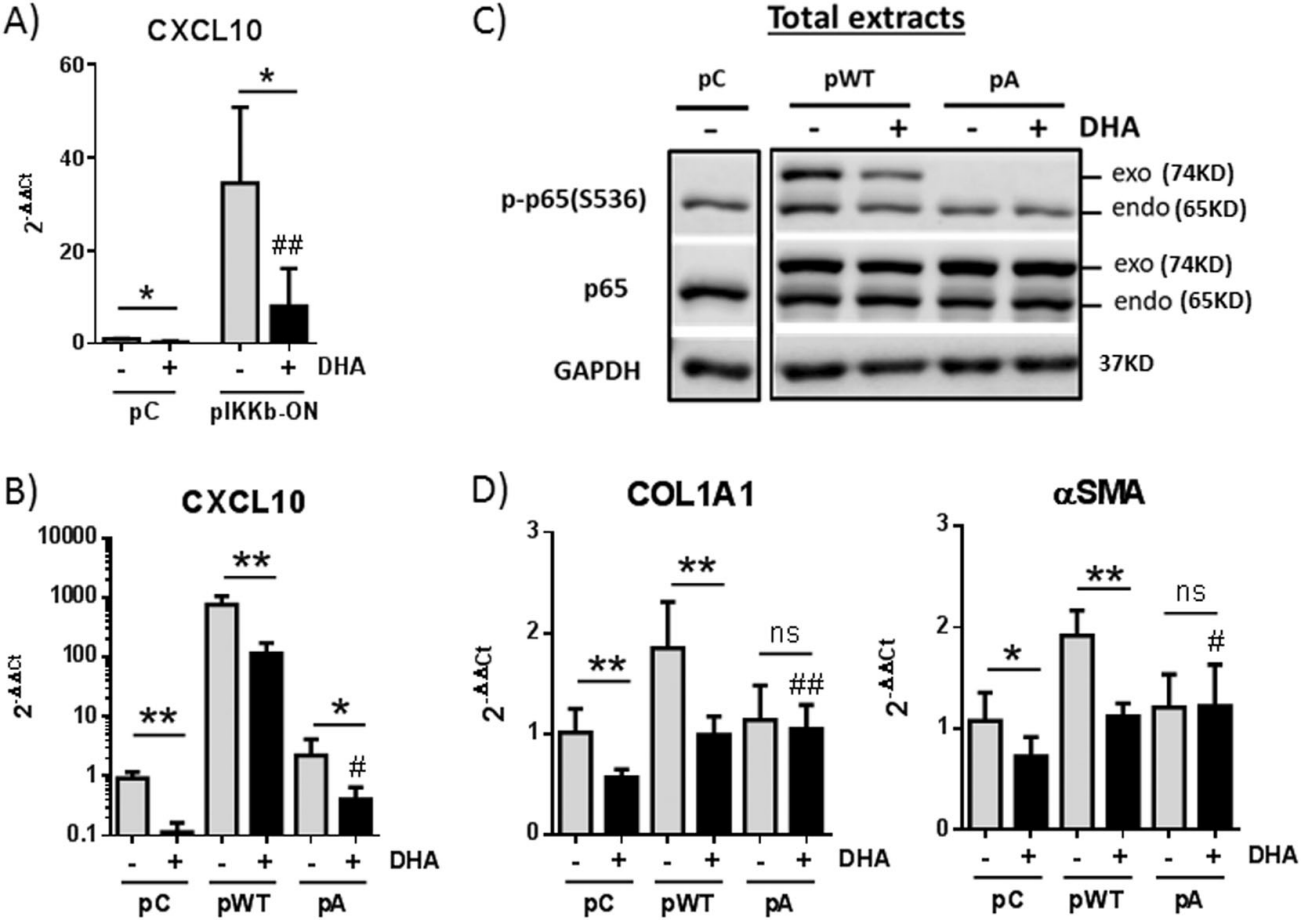
Phosphorylation of p65/RelA at S536 is essential for *CXCL10* induction and displays pro-fibrogenic effects in Lx2 cells. **a** *CXCL10* expression in cells transfected with a control plasmid (pC) or a plasmid expressing the constitutive active form of IKKβ (pIKKb-ON) and mock treated or treated with 30 µM DHA for 24 h. **b**–**d** Lx2 cells were transfected with a control plasmid (pC), a plasmid expressing wild-type p65/RelA (pWT) or a mutant S536A that cannot be phosphorylated (pA). At 24 h, the cells were mock treated or treated with 30 µM DHA for 24 h. *CXCL10* (**b**) and *COL1A1* and *αSMA* expression (**d**). Data are means ± SD from three experiments. p65/RelA and p-p65/RelA(S536) protein in total extracts (**c**)

The phosphorylation of p65(S536) can be mediated by several kinases, including IKKβ^17^. Although it has been described that DHA suppresses IKKβ phosphorylation and activity^18^, this is not the case in HSCs. Lx2 cells transfected with a plasmid that expresses a constitutively active IKKβ (pIKKB-ON) showed an increase in the levels of *CXCL10* mRNA and other pro-inflammatory transcripts compared to control cells (Fig. 7a and data not shown). Surprisingly, despite the strong activation of the NF-κB pathway obtained under constant IKKβ activation, DHA treatment blocked the induction of these pro-inflammatory genes. Similar results were observed in Lx2 cells transfected with a plasmid expressing wild-type p65/RelA (Fig. 7b). Expression of a mutant S536A p65/RelA that cannot be phosphorylated does not induce *CXCL10* levels but can diminish the inhibitory effect of DHA. This indicates that the regulation of p65(S536) phosphorylation by DHA is required to diminish the transcription of target genes. In agreement, western blot analyses indicated that overexpressed wild-type and mutant p65/RelA versions are efficiently transported to the nucleus of HSCs and that DHA can dephosphorylate exogenous wild-type p65/RelA (Fig. 7c and data not shown). Interestingly, DHA-mediated regulation of p65/RelA affects the expression of pro-fibrogenic factors, as the levels of *COL1A1* and *αSMA* transcripts increase in HSCs that overexpress wild-type but not S536A mutant p65/RelA (Fig. 7d). DHA treatment decreases the levels of phosphorylated p65(S536) and results in lower levels of pro-fibrogenic factors. Altogether, our results indicate that DHA inhibits the NF-κB pathway by decreasing p65(S536) phosphorylation even under conditions of strong pathway induction by constant IKKβ activation or by the overexpression of wild-type p65/RelA. In addition, p65/RelA activity contributes to the expression of pro-fibrogenic factors in HSCs.

## Discussion

One of the relevant findings of the present study was the presence of a profound DHA deficiency in the liver of cirrhotic patients. Notably, as liver cirrhosis progresses, liver depletion of DHA worsens in such a manner that Child-Pugh C patients have about half of the values found in normal hepatic tissue. The reasons for this defect could be diverse. ω3-PUFAs cannot be synthesized by mammals and need to be obtained from the diet. Thus, although a deficient diet may be a potential cause of low liver DHA content, other pathogenic factors, including malabsorption, changes in intestinal microbiota and the presence of porto-systemic shunts, may enter into play. Indeed, in liver cirrhosis, the intestinal barrier is disturbed and the composition of gut microbiota is deeply altered the dysbiosis being more intense as the disease progresses^19^. It seems possible that the pro-inflammatory milieu in the gut wall and the overgrowth of pathogenic bacteria may compromise DHA bioavailability. Interestingly, it has been shown that DHA contributes to protecting the integrity of the intestinal wall and restoring gut microbiota composition^20^. This is a relevant observation as dysbiosis has been implicated in the pathophysiology of liver cirrhosis^19,21^. On the other hand, a deficiency of ω3-PUFA may enhance microbiota alteration. Another important factor contributing to liver depletion of DHA is the presence of porto-systemic shunts in patients with advanced liver cirrhosis and portal hypertension as the resulting reduction in portal blood flow to the liver may reduce hepatic inflow of crucial nutrients including DHA.

In cirrhotic patients, DHA depletion may have a significant impact on liver tissue homeostasis, likely favouring fibrosis progression. Indeed, our data in mouse models of acute CCl_4_-induced liver damage indicate that DHA does not reduce hepatocellular damage while it strongly attenuates HSC activation and *αSMA* and *COL1A1* expression. The anti-fibrogenic effects of DHA have also been shown in animal models of chronic liver damage^14,16^, and in line with these findings it has been reported that DHA dampens the response of HSCs to TGFβ^14^. However, the molecular basis underlying these effects has not been characterized.

Our transcriptional analysis of primary mHSCs and Lx2 cells showed that DHA inhibits TGFβ and NF-κB pathways while inducing NRF2-dependent antioxidant response and the ATF4 branch of the UPR (Fig. 4). Regarding the last two biological processes, previous studies have shown that DHA promotes nuclear accumulation of NRF2, a factor that opposes TGFβ signaling by binding to SMADs and inhibiting transactivation^22–26^. Further, DHA has also been reported to cause ER stress and eIF2α phosphorylation^27^, and this effect may stimulate fibrogenesis^28–30^. However, in experiments directed to blocking or enhancing these two pathways we found that neither NRF2 nor ATF4 mediates in a significant manner the suppressive effect of DHA on *COL1A1* and *αSMA* expression by HSCs. Thus, it seems plausible that DHA may deploy its anti-fibrogenic activity by interfering with TGFβ and NF-κB signaling.

TGFβ is a potent inducer of pro-fibrogenic genes, such as *COL1A1*, *αSMA* and *PDGFRβ*, in HSCs. Moreover, TGFβ represses *ADRP*, a molecule that favors HSC quiescence and promotes lipid droplet formation by allowing the liberation of lipid drops from the ER membrane^31,32^. Our transcriptome analysis indicated that DHA inhibited the TGFβ pathway, attenuated fibrogenesis and reverted HSC activation (Fig. 4a, b). In fact, one of the key genes upregulated by DHA was *ADRP*. Accordingly, Lx2 cells treated with DHA were found to accumulate lipid drops in the cytoplasm, a characteristic feature of quiescent HSCs (Fig. 3d).

TGFβ functions by the activation and nuclear translocation of the SMAD2/3/4 factors. Then, SMADs bind the SBE (SMAD-binding element) sequence and allow the transcription of target genes. Several of these are induced very rapidly after stimulation with TGFβ. These early genes have been suggested to be pure SMAD responders^33^. The initial TGFβ response is followed by a secondary wave of transcription that may require co-operation of SMADs with other transcription factors such as STAT3, which help collagen expression^34^. Interestingly, we found that in HSCs DHA does not alter the initial transcriptional response to TGFβ but decreases the expression of the genes that are induced at 24 h. This observation was paralleled by changes in SMAD3 content in the cell nuclei as its values were comparable to controls at 6 h but markedly reduced at 24 h. This late nuclear depletion of SMAD3 by DHA causes the repression of pro-fibrogenic factors such as *COL1A1* or *αSMA* (late genes) without affecting the levels of the TGFβ inhibitor *SMAD7* (early gene). It should be noted that SMAD7 plays a critical role as an anti-fibrogenic factor by binding to the TGFβ receptor and blocking the association, phosphorylation and activation of the SMAD2/SMAD3 complex^35^.

In addition to TGFβ-induced pro-fibrogenic factors, activated HSCs (aHSCs) also secrete and sense inflammatory mediators. In fact, the NF-κB pathway is required for activation and survival of HSCs and for increasing the sensitivity of these cells to TGFβ^36^. In aHSCs, TNFα, angiotensin II, and other factors trigger IKKβ, which phosphorylates p65(S536). This transcription factor leads to the expression of pro-survival molecules and fresh angiotensin II, required to maintain a positive regulatory loop that guarantees aHSC survival^37^. The loop results in the constitutive activation of IKKβ and constitutive levels of p-p65(S536), characteristic of aHSCs. Increased apoptosis of aHSCs is observed when this pathway is blocked by antagonists of angiotensin receptors or inhibitors of IKKβ^37,38^. Here we show that DHA blocks this pathway by decreasing p65(S536) phosphorylation in an IKKβ-independent manner (Figs. 6 and 7). This is different from what has been described in most cells, where DHA decreases the activity of IKKβ and/or reduces the phosphorylation and degradation of the negative regulator IκBα, thus hindering nuclear translocation of NF-κB^39–42^. By decreasing p65(S536) phosphorylation, DHA blocks fibrosis development in different manners, including reduced production of pro-fibrogenic factors (p65 phosphorylation in S536 is required for *COL1A1* and *αSMA* expression by aHSC) and induction of aHSC apoptosis (p65 phosphorylation in S536 is an essential survival factor). Finally, DHA acting on HSCs also decreases inflammation by reducing the secretion of factors such as CXCL10, a pro-inflammatory chemokine that recruits leukocytes after liver injury and contributes to aHSC survival, migration and liver fibrosis development^43,44^.

As mentioned, DHA has demonstrated benefits in different animal models of liver damage^12–15^. These data stimulated clinical trials using ω3-PUFAs (EPA or combinations of DHA and EPA) in patients with non-alcoholic fatty liver disease (NAFLD) or NASH. These studies have shown positive effects on hepatic steatosis but have not demonstrated significant and reproducible effects on fibrosis^45–52^. It should be noted that these clinical trials have used a mix of ω3-PUFAs at doses lower than those applied in animal studies. As DHA shows higher anti-fibrogenic activity than other ω3-PUFAs, it seems possible that using pure DHA at higher doses could be necessary to obtain anti-fibrogenic effects in patients with liver disease. Also, combinations of DHA and therapies aimed at restoring gut microbiota could afford better therapeutic results.

## Materials and methods

### Human samples, animal models, serum biochemistry and immunohistochemistry

Human liver samples (Supplementary Table 1) and data from patients with or without alcoholic cirrhosis were provided by the Biobank of the University of Navarra and were processed following standard operating procedures approved by the Ethical and Scientific Committees. Animal studies were performed following the regulations of the Animal Care Ethical Committee from the University of Navarra. Mice were housed 4–6 per cage in a facility in which temperature (21 °C), humidity and light cycle (12 h light/dark) were controlled and had ad libitum access to food and water. C57 male mice (8 weeks old) were obtained from Harlan (Oxon, UK). For CCl_4_-induced chronic liver damage two independent experiments were performed. Liver fibrosis was developed by intraperitoneal (IP) injection of 0.75 µl/g CCl_4_ (Merck) twice per week for 4 (*n* = 4) or 6 (*n* = 4) weeks. For TAA-induced chronic liver damage, mice received IP injections of 200 mg/kg TAA, 3 per week for 8 weeks (*n* = 7). Fibrosis was confirmed in all animals by Sirius red staining and by evaluation of *COL1A1* and *αSMA* mRNA levels. Acute liver injury was induced with two doses of 1 µl/g CCl_4_ (2:5, v/v in sesame oil (Sigma S3547)) administered by IP injection (Fig. 2a). DHA treatment was performed by daily intragastric administration of 450 mg/kg DHANua (Nua Biological Innovations S.L) diluted 1:3, v/v in sesame oil and starting from 24 h after the first dose of CCl_4_. Similar results were observed when, instead of sesame oil, DHA was mixed thoroughly with phosphate buffered saline (PBS) to form an emulsion. Serum and liver samples were collected for further analyses. Serum aspartate transaminase (AST) and alanine transaminase (ALT) levels were determined (ABX diagnostics) in a Hitachi autoanalyzer (Roche). Liver tissues were fixed overnight in formaldehyde (252931.1211 Panreac), dehydrated in ethanol, and embedded in paraffin according to standard procedures. Sections were prepared and stained with a primary antibody against αSMA (Sigma, A2547).

### PUFA determination in human and mouse livers by LC-MS/MS

Liver tissue (25 mg) was homogenized in 100 μl of PBS with 0.01% BHT (2,6-di-tert-butyl-4-methylphenol, Merck Ref. 822021). Protein concentration was determined using the Bradford assay (Bio-Rad Ref. 500–0006). Lipid extraction was carried out following the protocol described by Bligh and Dyer^53^. DHA and AA were quantified using multiple reaction monitoring performed in a linear ion trap triple quadrupole mass spectrometer (QTrap 4000; Sciex, Concord, Ontario, Canada) coupled with an electrospray ionization source to an Ekspert UltraHPLC 100 (Eksigent, Dublin, CA, USA) with a Spherisorb ODS2 column (4.6 mm × 250 mm × 5 μm) (Waters). The instrument control, data acquisition, and lipid mediator quantification were performed using Analyst 1.5.2 software (Sciex). Mass spectrometry was carried out in negative ion mode using specific transitions 327/287 and 327/229. Calibration curves were constructed with an internal standard 332/288 and *r* values of curves were > 0.99 in all cases. DHA and AA (Sigma D2534 and A9673) were used as standard in the LC-MS/MS.

### Cell culture

Cells were grown at 37 °C in a 5% CO_2_ atmosphere. Human Lx2 cell line was kindly provided by S. Friedman (Mount Sinai hospital, NY) and cultured in Dulbecco's Modified Eagle's Medium supplemented with 2 mM glutamine, 100 μg/ml penicillin/streptomycin and 2% fetal bovine serum (FBS). Primary mHSCs were isolated from 16-week-old C57 healthy mice as described^54^. Briefly, mouse livers were digested with Pronase E (Sigma 000000010165921001) and Collagenase P (Sigma C5138) using a perfusion system. A Nycodenz (Sigma D2158) density gradient was used to separate other liver cells from HSCs, which have low density due to their high lipid content. Vitamin A autofluorescence and fat-storing characteristics were used to certify that the purity of the HSC population was higher than 95%. Primary mHSCs were cultured in DMEN-F12, 10% SFB, 100 μg/ml penicillin/streptomicin and 1% fungizone. The media was replaced every other day. When indicated, cells were incubated with TGFβ (RyD 240-B-002), TNFα (Prepotech 300-01 A) or the following fatty acids resuspended in DMSO (Sigma D4540): DHA (Sigma D2534), DPA (SC-200786), EPA (Sigma E2011) and palmitic acid (Sigma P5585). After resuspension in DMSO, the proper dose of fatty acids was mixed thoroughly with cell media containing FBS and added to the cells.

### Cell proliferation assays

Lx2 cells were plated in 96-well plates at 4000 cells/well, and the following day the treatment to be tested was added in a final volume of 100 μl. Cell viability was measured after 24, 48 and 96 h using the MTT assay. Briefly, 10 μl of MTT stock solution (5 mg/ml) was added to each well and cells were incubated for 4 h at 37 °C in an atmosphere of 5% of O_2_. Then, the medium was removed, 100 μl of DMSO-Isopropanol (1:1, v/v) was added and the absorbance was measured at 540 and 630 nm. The latter was considered background and was subtracted from the 540-nm measurement.

### Oil red staining

Lx2 cells were plated on four-well chambers (LabTek) at 20000 cells/well and treated as described. The following day, cells were fixed with 10% paraformaldehyde (PFA) for 15 min, dehydrated with 1,2-propanediol (Sigma 398039) for 5 min, stained with pre-warmed Oil Red O solution (Sigma O1516) for 10 min at 60 °C and incubated with 85% 1,2-propanediol solution for 5 min. Then, preparations were rinsed twice in distilled water, treated with Gill’s or Mayer’s hematoxylin to stain nuclei and washed twice in water for 3 min.

### Plasmid transfections and luciferase reporter assays

For overexpression experiments, p65 and p65-S536A plasmids were kindly provided by Carl Sasaki^55^ and pIKKb-ON plasmid was obtained from Addgene (Ref. 11105). Lx2 cells were seeded 24 h prior transfection at 170,000 cells/well in six-well plates. Transfection was done with 0.6 μg total DNA mixed with 1.2 μl Lipofectamine 3000 following the manufacturer’s instructions (Invitrogen, New York, NY, USA). The media was replaced 24 h later and the overexpression was assessed by quantitative real-time polymerase chain reaction (qRT-PCR) and western blot. For luciferase reporter assays, SBE4-Luc (Addgene, 16495) and pRL-CMV (Promega) were used. Lx2 cells were seeded 24 h prior transfection at 75,000 cells/well in 12-well plates. Transfection was done with 0.5 μl Lipofectamine 3000 mixed with 0.05 μg CMV-Renilla and 0.2 μg pSBE-Luc. One day later cells were treated as described (DMSO, 30 μM DHA, 7.5 ng/ml TGFβ) for 24 h, and Renilla and Firefly luciferase activities were measured using the Dual Luciferase System (Promega) according to the manufacturer’s instructions in a Berthold Luminometer (Lumat LB 9507). The values obtained for Firefly luciferase were corrected for equal transfection efficiency with those for Renilla luciferase.

### RNA extraction and qRT-PCR

Total RNA from tissue or cells was isolated using the Maxwell® 16 LEV simply RNA Purification Kit (Promega) and reverse transcription was carried out with SuperScript II reverse transcriptase (Invitrogen). Messenger RNA levels were determined by qRT-PCR using the SYBR® Green Supermix Reagent (Bio-Rad). Gene expression was normalized to the *RPLP0* housekeeping gene and expressed as 2^-∆CT^ or 2^-∆∆CT^, when several experiments were grouped. All primer sequences are listed in Supplementary Table 2.

### Expression arrays from Lx2 and primary mouse HSCs

Lx2 cells were treated with DMSO, 50 µM palmitic acid or 50 µM DHA for 48 h. Primary mHSCs were isolated from mice, cultured in six-well plates for 3 days and treated with DMSO or 100 µM DHA for 48 h. Total RNA was isolated from three independent samples using the Maxwell® 16 LEV simply RNA Purification Kit (Promega), and RNA quantity and quality were determined using a nanodrop (Thermo Scientific, Waltham, MA, USA) and Bioanalyzer (Agilent Inc., Santa Clara, CA, USA). Excellent quality samples were hybridized to the Human gene 2.0 (Affymetrix) or the GeneChip® Mouse Gene 2.0 (Affymetrix). The methods used for sample preparation, hybridization and data analysis were based on the Affymetrix GeneChip Expression Analysis Manual (Affymetrix, Santa Clara, CA, USA). Microarray data normalization was performed using the quantile algorithm and analyzed as described^56^. The biological knowledge extraction was complemented using the Ingenuity Pathway Analysis (Ingenuity Systems, www.ingenuity.com). Differentially expressed genes (B > 0) were scrutinized to identify the altered top canonical pathways and the top upstream regulators. All transcriptome data are available at the NCBI Gene Expression Omnibus (GEO) data repository (http://www.ncbi.nlm.nih.gov/geo).

### Protein extraction and western blot analyses

For nuclear/cytoplasmic fractionation, cells were suspended in 200 μl of Levrero buffer (50 mM Tris HCl (pH 7.5), 1 mM EDTA, 1% NP-40) containing protease inhibitor cocktail (Roche) and phosphatase inhibitors (10 nM NaF and 10 nM NaVO4) and incubated in ice for 10 min. Nuclei were separated from the cytosol by centrifugation at 3000 rpm. Nuclei and total cells were lysed in radioimmunoprecipitation assay (RIPA) buffer with protease and phosphatase inhibitors. For western bBlot analyses, around 10–20 μg protein was loaded per lane of polyacrylamide gel. After electrophoresis, proteins were transferred to polyvinylidene difluoride (PVDF) membranes using the Bio-Rad TransBlot (Bio-Rad, CA, USA). The membranes were blocked with 1% dried milk + 4% BSA in PBS and were incubated with the following primary antibodies: anti-GAPDH (2118), anti-SMAD3 (9523), anti-phospho-SMAD3 (9520), anti-p65 (8242), anti-phospho-p65-S536 (3033), anti-IKKB (2684), anti-IKKα (2682) and anti-phospho-EIF2α (9721) from Cell Signaling; anti-NRF2 (ab62352) from Abcam and anti-EIF2α (sc-11386), anti-HDAC2 (sc-7899), anti-IKBα (sc-371), anti-IKBβ (sc-9130), anti-p50 (sc-7178) and anti-C23 (sc-515312) from Santa Cruz Biotechnology. Primary antibodies were detected with the corresponding peroxidase-linked secondary antibodies, anti-rabbit IgG (7074, Cell Signaling) and anti-Mouse IgG (A0168, Sigma). Signal was developed with ECL-plus or ECL-ultra (Amersham) using the Odissey Li-Cor Hardware. Densitometry of the signal was carried out using Image Studio Lite v5.0 Software (LI-COR).

### Statistical analysis

Statistical analyses were performed using Prism 5 (GraphPad Software). Differences between two groups were analyzed using Student’s *t* test or U–Mann Whitney, whereas differences between three or more groups were analyzed using the Kruskal–Wallis ANOVA test followed by Dunn’s multiple comparisons test. MTT assays were analyzed by two-way ANOVA followed by Bonferroni’s multiple comparisons test. Results are represented as mean ± SD. Statistical significance is indicated by ***(*p* < 0.001), **(*p* < 0.01) or *(*p* < 0.05). ns indicates non-significant differences.

## Supplementary information

Supp Material and methods

Supplementary Tables

Supp. Fig. 1

Supp. Fig. 2

Supp. Fig. 3

Supp. Fig. 4
