# Supplementary material for: The cirrhotic liver is depleted of docosahexaenoic acid (DHA), a key modulator of NF-κB and TGFβ pathways in hepatic stellate cells
Source: Cell Death Dis. 2019 Jan 8;10(1):14. doi: 10.1038/s41419-018-1243-0 (PMC6325107; doi:10.1038/s41419-018-1243-0)
Supplement: Supplementary file 1 — Supp Material and methods [file 41419_2018_1243_MOESM1_ESM.docx]

**SUPPLEMENTARY MATERIAL AND METHODS**

**Cell treatments**

Lx2 cells were treated as indicated with ISRIB (Sigma SML0843) or Salubrinal (Sigma SML0951). Overexpression of NRF2 was carried out by transfecting the pNRF2 plasmid (pcDNA3-EGFP-C4-Nrf2; Addgene 21549). Inhibition was performed by transfecting siScramble1 (Sigma) or siNRF2-5′-GUAAGAAGCCAGAUGUUAATT.

**Supplementary Figure Legends**

**Supplementary Fig. 1. DPA and AA levels do not change in cirrhotic livers.** DPA and AA levels were determined by LC-MS/MS in liver samples from healthy and cirrhotic patients. The results show combined data from two independent experiments. Statistical significance was determined by Student's t-Test (ns, not significant).

**Supplementary Fig. 2. DHA treatment displays anti-fibrogenic and anti-proliferative effects in tissue culture.** a - b. Lx2 cells were treated with DMSO as a control, or the indicated doses of DHA and αSMA, COL1A1 and PDGFRβ mRNAs were evaluated by qRT-PCR using RPLP0 as a reference (a) and cell proliferation was measured by MTT assay (b). A representative result is shown out of 2 (b) or 4 (a) experiments performed. Statistical analyses used Kruskal-Wallis test followed by Dunn’s multiple comparisons test or two-way ANOVA followed by Bonferroni’s multiple comparisons test (*** p ˂ 0.001; ** p ˂ 0.01; * p ˂ 0.05; ns, not significant).

**Supplementary Fig. 3. Validation of the results obtained after microarray analysis.** Lx2 cells were treated with DMSO (Control), or 50µM of DHA (DHA) for 48h (n=3). RNA isolated from these cells was used to evaluate the expression levels of the indicated genes by qRT-PCR. RPLP0 was used as a reference in the qRT-PCR. Statistical significance was determined by Mann–Whitney U-test (*** p ˂ 0.001, ** p ˂ 0.01, * p ˂ 0.05).

**Supplementary Fig. 4. ATF4 and NRF2 do not contribute to the DHA anti-fibrogenic effects. a - b.** Cells were transfected with a control plasmid (pC), a plasmid overexpressing NRF2 (pNRF2) (a), a control siRNA or a siRNA targeting NRF2 (b). Thirty hours after transfection, the cells were incubated with DMSO or 50µM DHA for 30 additional hours. HMOX1, COL1A1 and αSMA mRNA levels were quantified by qRT-PCR (a, b). **c –d.** Cells were treated with DMSO or with 25 µM of Salubrinal (Sal) for 30 h (c) or with DMSO, 50µM DHA, 200 nM of ISRIB, or the same doses of DHA and ISRIB for 30 h (d). TRIB3, COL1A1 and αSMA expression was quantified by qRT-PCR (c, d). RPLP0 was used as a reference in the qRT-PCR. Statistical significance was determined by Mann–Whitney U-test (*** p ˂ 0.001, ** p ˂ 0.01, * p ˂ 0.05, ns. not significant).
