# Supplementary material for: The cirrhotic liver is depleted of docosahexaenoic acid (DHA), a key modulator of NF-κB and TGFβ pathways in hepatic stellate cells
Source: Cell Death Dis. 2019 Jan 8;10(1):14. doi: 10.1038/s41419-018-1243-0 (PMC6325107; doi:10.1038/s41419-018-1243-0)
Supplement: Supplementary file 2 — Supplementary Tables [file 41419_2018_1243_MOESM2_ESM.docx]

**Supplementary Tables**

**Supplementary table 1. Human parameters.**

| Parameters | Healthy | Cirrhotic |
| --- | --- | --- |
| Male:Female | 8:4 | 11:3 |
| Age | 58.9 ± 11.9 | 58.3 ± 8.2 |
| BMI | 24.9 ± 3.9 | 27.1 ± 6.1 |
| Direct bilirubin (mg/dL) | 0.3 ± 0.1 | 2.0 ± 1.6 |
| Indirect bilirubin (mg/dL) | 0.3 ± 0.2 | 1.7 ± 1.2 |
| Total bilirubin (mg/dL) | 0.7 ± 0.5 | 3.8 ± 2.7 |
| ALP | 98.6 ± 67.0 | 101.2 ± 50 |
| GGT | 79.8 ± 61.2 | 48.1 ± 35.7 |
| s-ALT (GPT) | 30.4 ± 16.9 | 19.0 ± 9.3 |
| s-AST (GOT) | 23.9 ± 11.0 | 33.4 ± 22.8 |

**Data are expressed as mean ± SD**

**Supplementary table 2. Primer sequences for qRT-PCR.**

| Name | Primer forward (5'-3') | Primer reverse (5'-3') | Specie |
| --- | --- | --- | --- |
| RPLP0 | GGAGAAACTGCTGCCTCACA | TCGGGTCCTAGACCAGTGTT | mouse |
| RPLP0 | AGCCCAGAACACTGGTCTC | ACTCAGGATTTCAATGGTGCC | human |
| αSMA | AATGGCTCTGGGCTCTGTAA | TGGTGATGATGCCATGTTCT | human, mouse |
| Col1a1 | CAGATTGAGAACATCCGCAG | TCGCTTCCATACTCGAACTG | mouse |
| Col1a1 | GGCCCAGAAGAACTGGTACA | CGCTGTTCTTGCAGTGGTAG | human |
| ADRP | AGCAGGCTCTCAGCAGGG | GTACACCTTGGATGTTGG | human |
| ADRP | CCGCTTATGTCAGTACAAAGG | TTGCAGGCATAGGTATTGGC | mouse |
| PDGFRβ | AACCACCATTGGGGACAGGGA | CTGGCGGACCACAGTCTGCA | human, mouse |
| CTGF | ACCTGTGGGATGGGCATCTC | CGGATGCACTTTTTGCCCTTC | human, mouse |
| cMet | CTCGATCAGGACCATCAACC | TGACTGCAGGACTGGAAATG | human |
| TGFβ2 | ACCCCACATCTCCTGCTAATGT | GAAGGCAGCAATTATCCTGCACA | human, mouse |
| Serpine1 | GGCTTCATGCCCCACTTCTTC | CAGCACCAGGCGTGTCAGCT | human, mouse |
| SMAD7 | TTCCTCCGCTGAAACAGGG | CCTCCCAGTATGCCACCAC | human |
| Hmox1 | TGAAGGAGGCCACCAAGGAG | AAGGAGGCCATCACCAGCTT | human, mouse |
| Trib3 | TGCCCTACAGGCACTGAGTA | GTCCGAGTGAAAAAGGCGTA | human |
| CXCL10 | CTGTACGCTGTACCTGCATCA | TTCTTGATGGCCTTCGATTC | human |
| CXCL9 | TTTTCCTCTTGGGCATCATC | TTGGGGCAAATTGTTTAAGG | human |
| TNFα | CCCCAGGGACCTCTCTCTAA | CAGCTTGAGGGTTTGCTACA | human |
| HGF | TATGCAGAGGGACAAAGGAAA | AACAAAAGCCTTGCAAGTGAA | human |
| COL4A1 | TGCTTACAGCTTTTGGCTCGC | CGCAGCTCCCCTGCCTTCAA | human, mouse |
