# Supplementary figures and images for: The cirrhotic liver is depleted of docosahexaenoic acid (DHA), a key modulator of NF-κB and TGFβ pathways in hepatic stellate cells
Source: Cell Death Dis. 2019 Jan 8;10(1):14. doi: 10.1038/s41419-018-1243-0 (PMC6325107; doi:10.1038/s41419-018-1243-0)

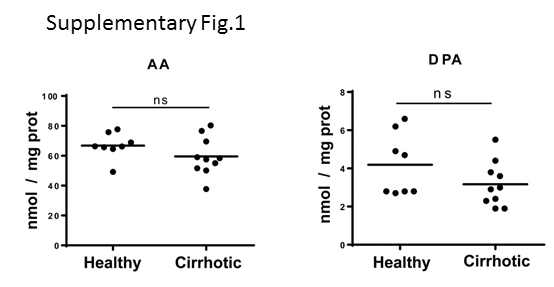

Supplement: Supplementary file 3 — Supp. Fig. 1 [file 41419_2018_1243_MOESM3_ESM.tif]

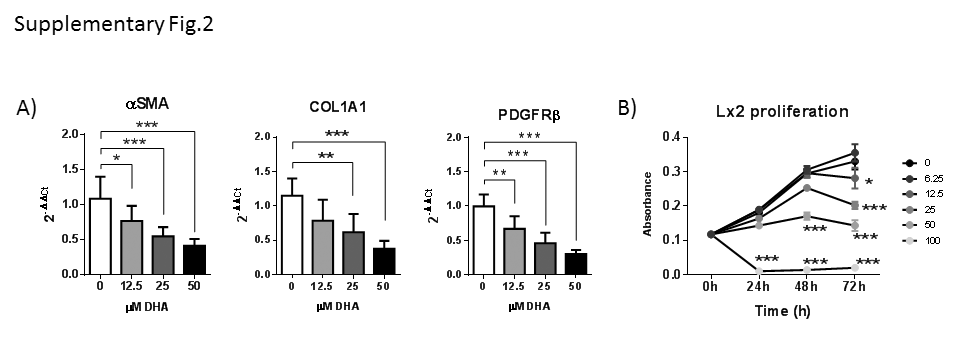

Supplement: Supplementary file 4 — Supp. Fig. 2 [file 41419_2018_1243_MOESM4_ESM.tif]

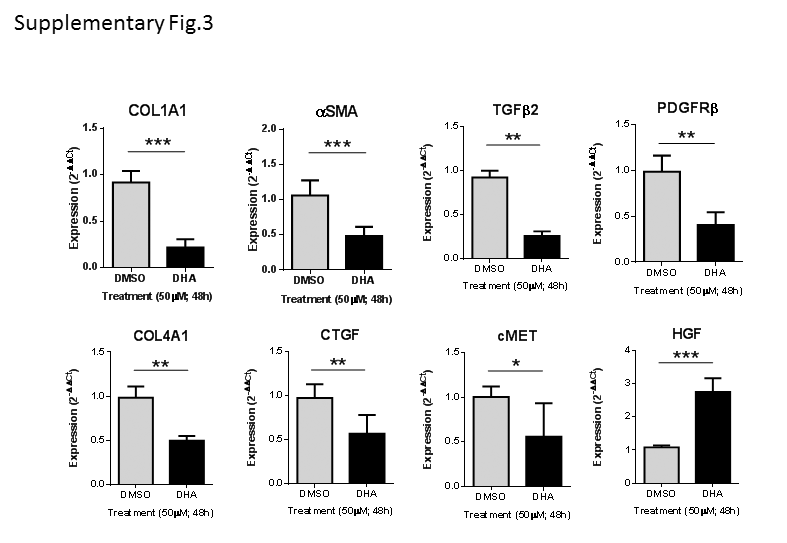

Supplement: Supplementary file 5 — Supp. Fig. 3 [file 41419_2018_1243_MOESM5_ESM.tif]

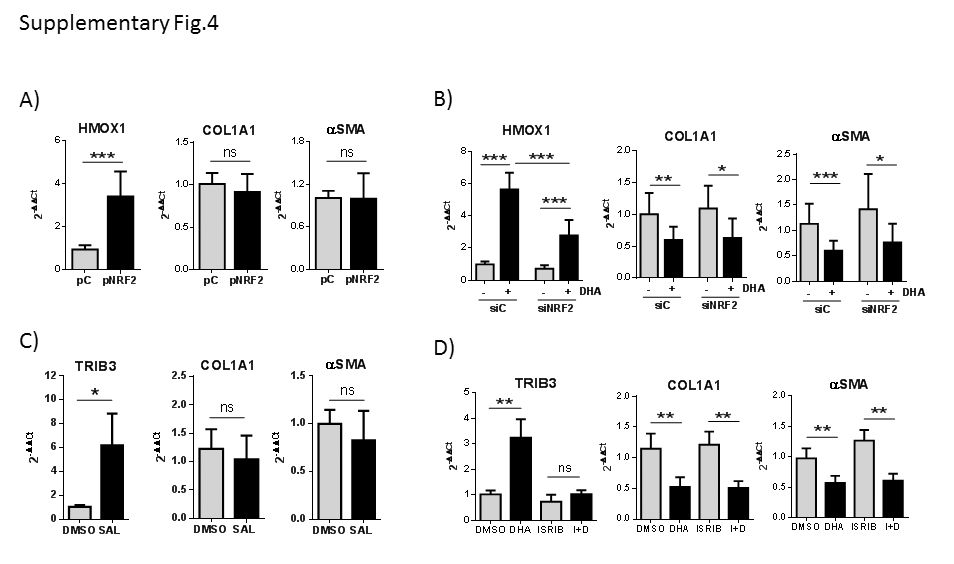

Supplement: Supplementary file 6 — Supp. Fig. 4 [file 41419_2018_1243_MOESM6_ESM.tif]
